# Disparity in cognitive factors related to cancer screening uptake based on the theory of planned behavior

**DOI:** 10.1186/s12885-024-12607-w

**Published:** 2024-07-16

**Authors:** Mehdi Mirzaei-Alavijeh, Mahin Amini, Mehdi Moradinazar, Mehdi Eivazi, Farzad Jalilian

**Affiliations:** 1 Social Development and Health Promotion Research Center, Health Institute, Kermanshah University of Medical Sciences, Kermanshah, Iran; 2 Research Center for Environmental Determinants of Health, Health Institute, Kermanshah University of Medical Sciences, Kermanshah, Iran; 3 Clinical Research Development Center, Motazedi Hospital, Kermanshah University of Medical Sciences, Kermanshah, Iran

**Keywords:** Neoplasms, Health inequities, Theory of planned behavior

## Abstract

**Introduction:**

Early detection of cancer is a highly effective way to decrease cancer-related deaths. The purpose of this study was to determine the disparity in cognitive factors related to cancer screening uptake based on the theory of planned behavior (TPB).

**Methods:**

In this cross-sectional study, conducted in Kermanshah County, the west of Iran, during 2019, a total of 1760 people aged 30 to 75 years old, were randomly selected to participate voluntarily in the study. Participants filled out a questionnaire including the socio demographic variables, socioeconomic status (SES), TPB variables, and cancer screening uptake behaviors.

**Results:**

The mean age of respondents was 45.28. 44.96% of the participants had undergone cancer screening at least once. Socioeconomic status (SES) and gender had the most significant impact on the disparity in cancer screening uptake, with contributions of 74.64% and 22.25% respectively. Women were 8.63 times more likely to be screened than men. Participants with a family history of cancer had a 2.84 times higher chance of being screened. Single individuals were significantly less likely to be screened compared to married individuals. The concentration index for attitude, subjective norms (SN), perceived behavior control (PBC), behavior intention, and cancer screening uptake was 0.0735, 0.113, 0.333, 0.067, and 0.132 respectively. Intention (Beta = 0.225 and P: < 0.001) is a significant predictor of cancer screening behaviors.

**Conclusion:**

The findings of this study are highly valuable for health policymakers in Iran. They emphasize the significance of creating, executing, and assessing campaigns that promote intention, PBC and SN, particularly among disadvantaged individuals. By doing so, we can effectively decrease the disparity in cancer screening rates. It is crucial to prioritize men, single individuals, and disadvantaged groups in cancer screening promotion programs. This knowledge can be utilized to develop an intervention that is guided by theory and supported by evidence, with the aim of increasing cancer screening rates and minimizing disparities.

## Introduction

Cancer is now a major contributor to early death worldwide [1]. Cancer-related death rates are higher in low- and middle-income countries compared to high-income countries; this is because of issues such as lack of early diagnosis, delayed diagnosis, and unequal access to healthcare services [2]. It is estimated that the number of cancer cases in low- and middle-income countries will increase five times by the year 2030 [3].

In Iran, cancer is the third leading cause of death after cardiovascular diseases and accidents; and approximately 135,000 new cases and 41,940 deaths are reported each year [4]. Skin, stomach, bladder, prostate, and colorectal cancers are the most common types of neoplasms in men in Iran, based on age-standardized rates; For women, the most common types are skin, colorectal, stomach, and esophageal cancers; Overall, the most frequent cancers in Iran are skin, stomach, colorectal, and bladder cancers [5].

The UN aims to reduce deaths related to non-communicable diseases by one-third by 2030 [6]. Early detection of cancer is proven to be highly effective in reducing cancer mortality [7]. In the past few decades, developing countries like Iran have faced challenges in ensuring equal access to healthcare services and effectively managing resources [3]. Iran now provides free population-based screening programs for three types of cancer: cervical (pap smear), breast (Mammography), and colon (Fecal Immunochemical Test -FIT); the Ministry of Health in Iran aims to offer opportunistic screening for additional types of cancer (Including other common cancers in Iran; such as skin, stomach, bladder, and prostate for at risk group [8].

People who understand the advantages of cancer screening or have a positive outlook on it prioritize cancer screening in their health plans [9]. Therefore, comprehending the factors that affect the uptake of cancer screening tests is crucial for developing and implementing timely and effective health care interventions; this information is valuable for health planners working on promoting programs for cancer screening and early detection [10]. Several cognitive factors, including attitude, intention, Perceived Behavior Control (PBC), self-efficacy, and subjective norms, play a significant role in influencing the likelihood of adopting or refusing a healthy behavior like cancer screening [11–13]. However, evidence suggests that there are also disparities in cognitive factors in cancer screening uptake [14]. Despite significant advancements in healthcare, there are still notable health disparities globally; many of these disparities can be linked to differences in Socioeconomic Status (SES), which is typically measured by social determinants of health like education, employment, and income [15, 16].

The purpose of this study was to determine the disparity in cognitive factors related to cancer screening uptake based on the theory of planned behavior.

## Materials and methods

### Participants and setting

This study was a cross-sectional study that conducted among the urban population of Kermanshah in 2019. Sampling was done in several steps based on the following steps; first, the city of Kermanshah was divided into 8 regions according to the municipal areas. Then, each of the urban areas divided into 10 blocks, and 2 blocks were randomly selected and eligible households included in the study. Data collection completed using a written questionnaire based on interviewing participants.

For data collection from participants, the interviewer went to the door of a house in the designated place and then moved to the right by standing back in the first household (head of the cluster) to complete the required number of samples. If access to the household within the cluster was not available, or the household was reluctant to participate in the study, or the household did not live beyond 30 years, the household was replaced. Data collection lasted for three months, from July to September 2019. Four trained public health experts conducted interviews and gathered data for this project. The data was gathered by conducting interviews (based on paper based survey) with participants.

### Sample size

The sample size was calculated at a 95% significant level, according to the prevalence rate of 0.5, the accuracy of 0.025, and considering the 10% attrition rate, a sample of 1760 was estimated. After removing incomplete questionnaires, 1668 questionnaires were analyzed (the response rate in the present study was 94.7%).

### Measure

The questionnaire included four sections including (socio demographic variables, SES, TPB variables, and cancer screening uptake behaviors).

#### Socio demographic variables

Socio demographic items were designed to gather information related to age (years), gender (male, female), marital status (single, married, divorced, deceased spouse), level of education (elementary, middle school, diploma, university), and history of cancer in the family (yes, no).

### Socio-economic status

The SES index as the main variable representing household economic status was calculated by using Principal Components Analysis (PCA) and taking into account the economic and social variables of the participants. The SES information was related to durable goods and social determinants including ownership of a car, refrigerator, television (s), separate freezers, a washing machine, vacuum cleaner, mobiles, bicycles, laptops, etc., as well as housing, number of rooms in the house, heating, air conditioner, domestic and foreign travel per year. These variables were entered into the PCA model. The study population was classified based on an SES variable with the following levels: poor, middle, and rich, and was used as an index for SES in disparity analysis.

### Theoretical Framework

The Theory of Planned Behavior (TPB) was developed by Icek Ajzen in 1985. According to the TPB, the main factors influencing future behavior are a person’s behavioral intentions and beliefs about their ability to control their behavior (perceived behavioral control - PBC). Intentions are influenced by three main factors: (a) attitudes, which represent a person’s feelings about the behavior; (b) subjective norms (SN), which represent the individual’s perception of what others think about the behavior; c) PBC This is a person’s belief that they have the ability to control their behavior. If PBC accurately reflects an individual’s control over their behavior, we would expect it to be able to directly predict that behavior [17].

In this study, we evaluated the face validity, content validity, and internal consistency of a questionnaire. To assess face validity, we conducted qualitative interviews with 12 experts and made adjustments to the questionnaire based on their feedback. The content validity of the TPB questionnaire was evaluated using both quantitative and qualitative methods. Another group of 12 experts provided feedback on the relevance, difficulty, and ambiguity of the items, which were then modified accordingly. Additionally, 12 different experts assessed the necessity of each item as “essential”, “useful but not essential”, or “not essential” to determine quantitative content validity. The minimum acceptable values for content validity index (CVI) and content validity ratio (CVR) were set at 0.79 and 0.62, respectively [18]. The expert panel consisted of health policy makers, nursing experts, public health experts, general practitioners, and experts in health education and promotion. Internal consistency was measured using Cronbach’s Coefficient Alpha for the TPB variables. Before the main study, a pilot study was conducted with 20 participants to test the questionnaire’s clarity, length, comprehensiveness, and completion time, as well as to estimate internal consistency. There were ten items that measured the four determinants of (a) attitude, (b) SN, (c) PBC, and (d) intention. Specifically, three items measured attitudes towards the cancer screening test uptake (e.g., uptake of cancer screening tests can reduce my chances of dying from cancer). There were two items that measured the SN encourage cancer screening test uptake (e.g., if I uptake cancer screening tests, my family will confirm it). Four items measured the PBC to cancer screening test uptake (e.g., I believe that I can decision-making to uptake the cancer screening test). The intention to cancer screening test uptake was measured by one item (e.g., I intend to uptake cancer screening test during the current year). A 5-point Likert-type scaling, ranging from 1 (strongly disagree) to 5 (strongly agree), was used. The reliability coefficients for the abovementioned constructs were as follows: attitude (α = 0.87); SN (α = 0.74), and PBC (α = 0.86), attesting to the internal consistency of the measures.

#### Cancer screening uptake behaviors

History of screening for bladder, lung (PET scan), skin (use of sunscreen), colorectal (FIT), prostate (Rectal exam and PSA), breast (mammography, breast self-examination, and clinical breast examination) and cervix (Pap smear) were evaluated as yes (1) and no (0). By summing up all these screenings, the cancer screening uptake behaviors variable was created. Finally, a score between 0 and 7 was created. Furthermore, the participants were split into two groups: those who had undergone at least one cancer screening test and those who had never been screened for cancer.

### Statistical analysis

The data analysis for this study was conducted using SPSS version 16 statistical software. Descriptive information, including frequency, percentage, mean, and Standard Deviation (SD), was used to summarize the data. The Independent Samples t Test was utilized to examine the relationship between cancer screening history and TPB variables. Additionally, the Pearson correlation coefficient was used to assess the correlation between different components of the TPB. Assumptions such as linearity and independency for quantitative outcomes were evaluated and confirmed. Multiple linear regression was performed to identify predictors of cancer screening test uptake (model 1). Variables with a p-value greater than 0.25 were excluded in the adjusted model, while variables with a p-value lower than 0.25 were retained (model 2). Cronbach’s coefficient alpha was calculated to assess the reliability of the data.

SES-related disparity was measured through the concentration index in TPB determinants and history of cancer screening uptake, in the entire population and for each of the independent variables. The numerical value of the concentration index is between − 1 and + 1 [19], which was measured by the following formula.

$$CI = \frac{2}{{\bar Y}}COV\left( {Yi.Ri} \right)$$

Where $\bar Y$ is the mean of health variable in the whole population, R_i_ is the fractional rank by income and Y_i_ is the health variable for individual i.

The concentration index is extracted from the concentration curve and it equals twice the space between the focus curve and the equality line (45-degree). If the index is zero, this means that the variable was distributed equally among socio-economic groups [20].

Using Decomposition Analysis, the net contribution of each factor in the total economic disparity in the outcome variable was quantified. The normalized concentration index (Cn = CI/1-µ) which is used for the two-mode outcome variable; It was decomposed according to the following formula [21].

$$Cn = \sum\nolimits_k {\left( {\frac{{{\beta _k}{{\bar x}_k}}}{\mu }} \right)} {C_k} + \frac{{\frac{{G{C_\varepsilon }}}{\mu }}}{{1 - \mu }}$$

The $\bar {X}$ represents mean of each of the investigated factors, C_K_ represents the concentration index value for the ${\rm X}$variable. Elasticity of each variable was calculated with a $\frac{{{\beta _k}{{\bar {x}}_k}}}{\mu }$ formula. ${\beta _k}$ is the marginal effect value for each variable. Ʃ $\:\left(\frac{{\beta\:}_{k}{\stackrel{-}{x}}_{k}}{\mu\:}\right){C}_{k}$ the participation share is absolute and shows the sum of the concentration index described by the variables under investigation. If all the variables under study cannot describe the value of the total concentration index in${{\rm X}_k}$; the remaining component is presented with $\frac{{\frac{{G{C_\varepsilon }}}{\mu }}}{{1 - \mu }}$ [21]. By dividing the absolute share of participation by the concentration index of the dependent variable for each factor, the percentage share of participation for that factor is obtained.

## Results

The mean age of respondents was 45.28 [95% CI: 44.75, 45.81], ranged from 30 to 75 years. 44.96% (750/1668) of the participants had a history of performing at least one cancer screening uptake. Table 1 shows more details regarding the participants’ socio-demographic variables.

**Table 1:** Distribution of the socio-demographic variables among the participants

Variables		Number	Percent
Age group (year)	30–39	624	37.4
	40–49	517	31
	50–59	298	17.9
	> 60	229	13.7
Gender	Men	761	45.6
	Women	907	54.4
Marital status	Married	1345	80.6
	Single	210	12.6
	Widow	113	6.8
Education level	Illiterate	144	8.6
	Primary	267	16
	Secondary	199	11.9
	High school	398	23.9
	Academic	660	39.6
History of cancer in family	No	1294	77.6
	Yes	374	22.4

Table 2 shows the concentration index analysis and odds ratio results for the history of cancer screening test uptake in the participants. As can see in Table 1, the SES with 74.64% had the largest contribution among all determinants in increasing disparity in cancer screening uptake. Gender was the next important factor in increasing disparity with 22.25%. Women were 8.63 (CI 95%: 6.87, 10.84) times more likely to be screened than men. The odds ratio of screening for single people was 0.72 (CI 95%: 0.53, 0.97), which shows that single people did screening significantly less than married people. On the other hand, widowed people had 1.71 (CI 95%: 1.16, 2.53) times more chance of screening than married people. Participants who reported a positive family history of cancer had a 2.84 times more chance of screening. The predictor variables accounted for 105.66% of the disparity in the cancer screening uptake.

**Table 2:** Concentration index analysis and odds ratio results for the history of cancer screening tests uptake in the participants

Variables		Proportion	Odds ratio (95%CI)	Ck^*^	Absolute Contribution^**^	Percent contribution^***^	Sum present contribution^****^
**Gender**	Women	0.544	8.63 (6.87–10.84)	0.056	0.029	22.248	22.25
**Age group (year)**	40–49	0.310	1.14 (0.91–1.45)	0.035	0.001	0.776	-9.95
	50–59	0.179	1.18 (0.89–1.55)	-0.076	-0.004	-2.658	
	> 60	0.137	0.98 (0.72–1.33)	-0.201	-0.011	-8.066	
**Marital status**	Single	0.126	0.72 (0.53–0.97)	0.023	-0.001	-0.716	-2.44
	Widow	0.068	1.71 (1.16–2.53)	-0.204	-0.002	-1.723	
**Education level**	Primary	0.160	0.71 (0.47–1.07)	-0.290	-0.001	-1.062	7.36
	Secondary	0.119	0.69 (0.45–1.06)	-0.287	-0.011	-8.588	
	High school	0.239	0.96 (0.66–1.41)	0.021	0.002	1.164	
	Academic	0.396	0.79 (0.55–1.13)	0.243	0.021	15.844	
**History of cancer in family**	Yes	0.224	2.84 (2.23–3.60)	0.119	0.018	13.800	13.80
**SES**	Middle	0.333	1.45 (1.14–1.84)	0.000	0.000	0.000	74.64
	Rich	0.333	1.83 (1.44–2.32)	0.667	0.099	74.642	
**Total explained**					0.140		105.66
**Residual**					0.008		5.66
**Total**					0.132		100

^*^CK is the value that represents the concentration index for the variable.

^**^Absolute contribution is the specific amount or value that a factor or variable contributes to overall disparity. It shows the direct impact of that factor on the level of disparity.

^***^Percent contribution indicates the proportion or percentage of the total disparity that can be attributed to a specific factor or variable. It helps to understand the relative importance of each factor in contributing to the overall disparity.

^****^Sum present contribution in disparity refers to the total combined contribution of all factors or variables to the overall level of disparity. It represents the cumulative impact of various factors on the observed disparity.

**Fig. 1:**
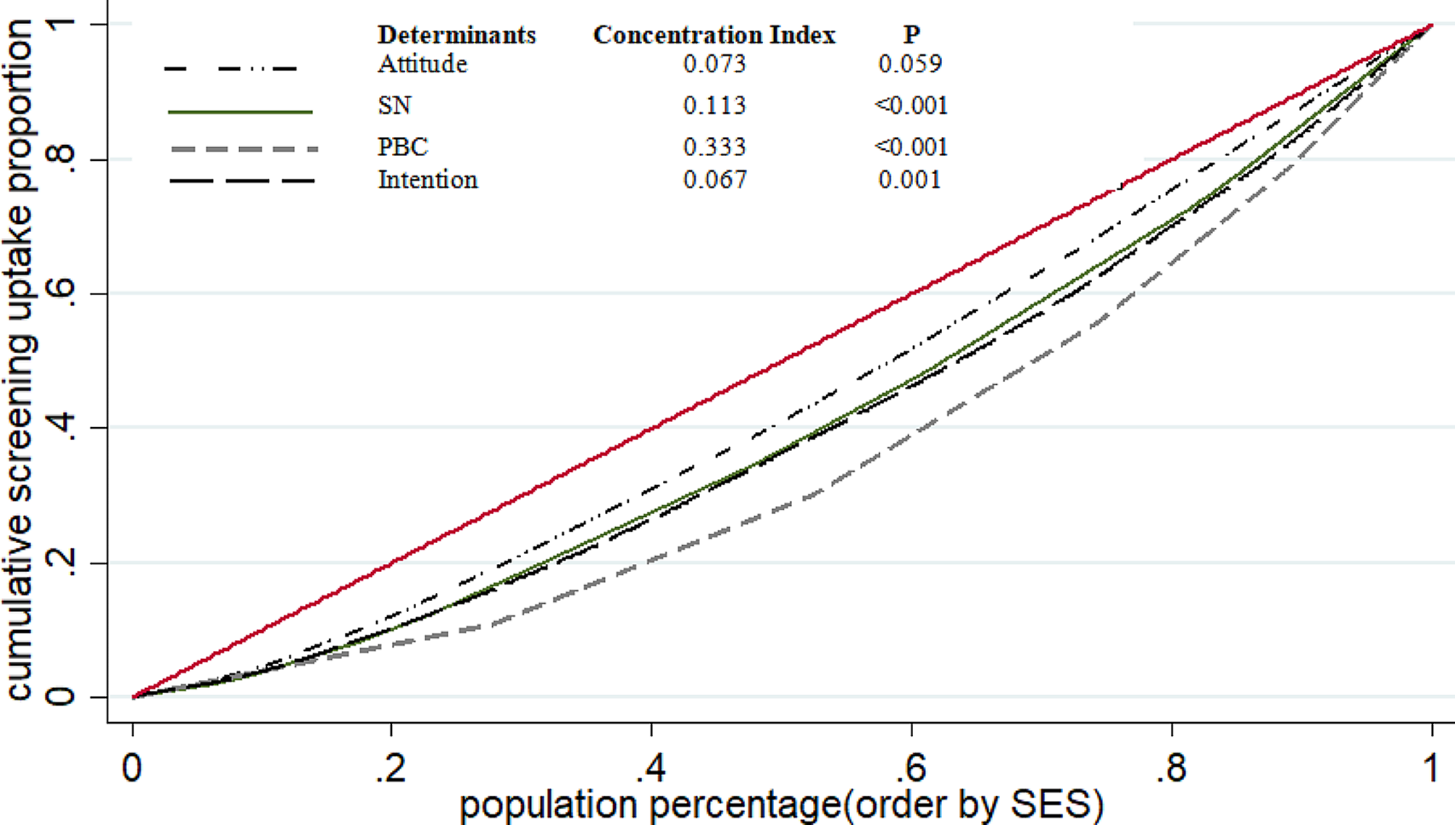
The concentration curves for TPB variables

The concentration index for attitude, SN, PBC, and intention were 0.0735 (P: 0.059), 0.113 (*P* < 0.001), 0.333 (*P* < 0.001), and 0.067 (P: 0.001), respectively. That indicating that the TPB determinants (especially in PBC) concentration was greater in subjects with a higher SES. The overall concentration index for TPB determinants is shown in Fig. 1. The results indicate that not only is there disparity in access to cancer screening, but there is also disparity in TPB variables. This disparity is most pronounced in PBC. This indicated that individuals in higher socioeconomic groups have a more positive attitude, SN, PBC, and intention to undergo cancer screening.

**Fig. 2:**
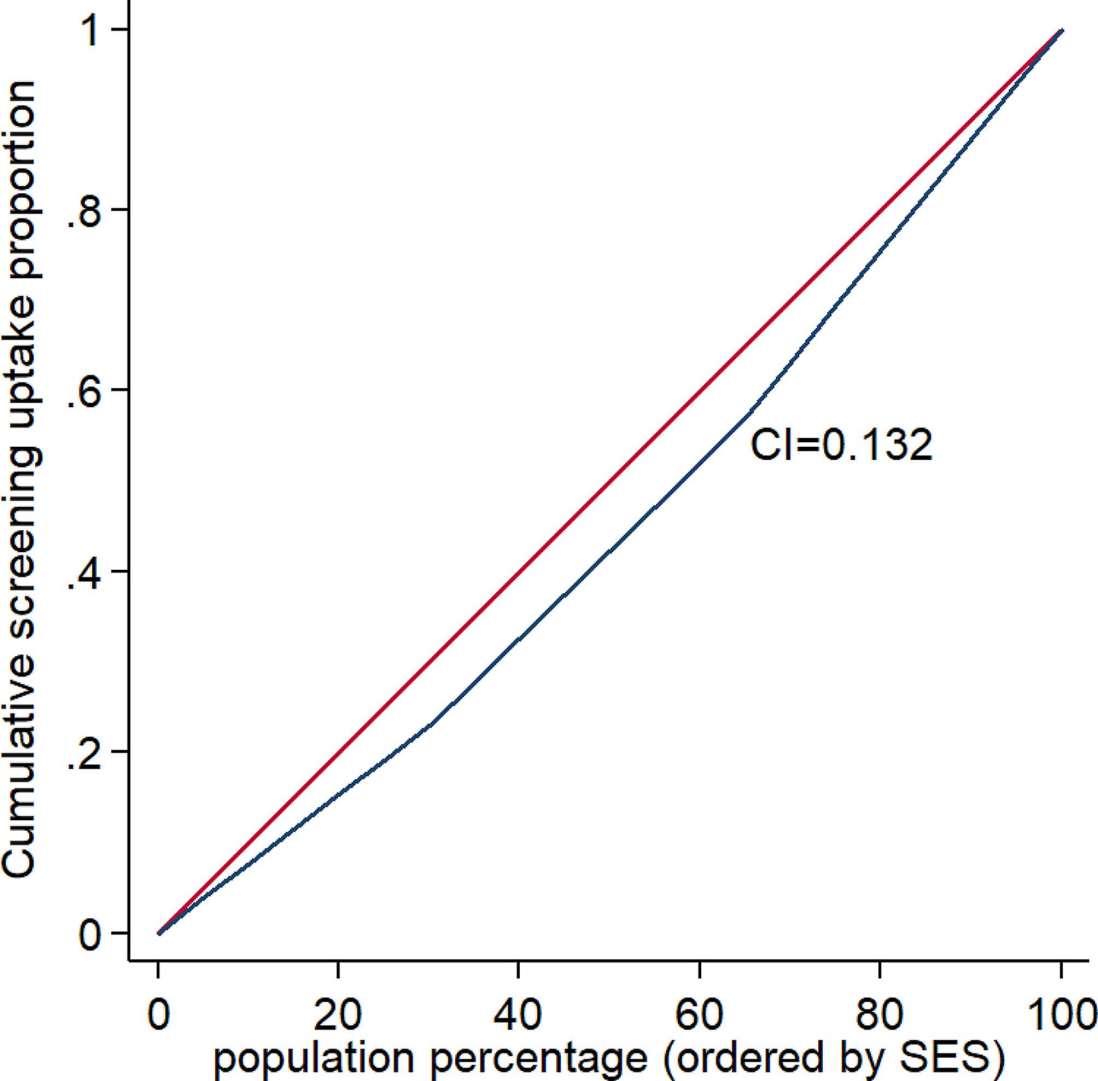
The concentration curves for cancer screening test uptake in participants

Moreover, the overall concentration index for cancer screening test uptake is shown in Fig. 2. As seen in Fig. 2, the overall concentration index for cancer screening test uptake was 0.132 (*P* < 0.001), which indicated that the cancer screening test uptake concentration was greater in participants with a higher SES.

Table No. 3 displays the history of cancer screening test uptake and TPB variables. The results indicate that the scores for SN, PBC, and intention are significantly higher among participants who have at least one screening test. However, the average score for attitude is not significantly different between those who have completed at least one screening test and those who have not. Furthermore, the correlation between different determinants of TPB was also shown in Table 3. The results showed that intention to cancer screening test uptake was correlated with the positive attitudes towards the cancer screening test uptake (*r* = 0.375), SN encourage cancer screening test uptake (*r* = 0.495), and PBC of the cancer screening test uptake (*r* = 0.521).

**Table 3:** History of cancer screening tests uptake and TPB variables

TPB determinants	History of cancer screening tests uptake	Mean (SD)	*P*	X1	X2	X3	X4
X1. Attitude	No (*n* = 918)	12.14 (2.85)	0.727	1			
	Yes (*n* = 750)	12.19 (3.02)					
X2. SN	No (*n* = 918)	6.54 (2.11)	0.001	0.514^**^	1		
	Yes (*n* = 750)	6.90 (2.30)					
X3. PBC	No (*n* = 918)	11.89 (4.22)	0.006	0.437^**^	0.722^**^	1	
	Yes (*n* = 750)	12.48 (4.37)					
X4. Intention	No (*n* = 918)	2.32 (1.26)	< 0.001	0.375^**^	0.495^**^	0.521^**^	1
	Yes (*n* = 750)	2.85 (1.33)					

^**^Correlation is significant at the 0.01 level (2-tailed)

The Table 4 displays the factors that influence cancer screening participation according to TPB variables. Initially, a Crude analysis was conducted. The results of this analysis indicated that all TPB variables should be considered in the final model. The adjusted analysis results are also included in Table 4. These results reveal that intention (Beta = 0.225 and P: < 0.001) is a significant predictor of cancer screening behaviors.

**Table 4:** Predictors of the cancer screening uptake behaviors based on TPB variables

	Model 1 (Crude)				Model 2 (Adjusted)			
	B	Std. Error	Beta	P	B	Std. Error	Beta	P
Attitude	0.015	0.009	0.041	0.097	0.013	0.010	0.035	0.219
SN	0.040	0.012	0.082	0.001	0.011	0.018	0.023	0.544
PBC	0.018	0.006	0.072	0.003	0.011	0.009	0.046	0.202
Intention	0.159	0.019	0.199	< 0.001	0.180	0.023	0.225	< 0.001

## Discussion

The main aim of the present study was to determine the disparity in cognitive factors related to cancer screening based on the theory of planned behavior. As the findings show, disparity is observed in the TPB determinant. Thus, the rich group has a better attitude, SN, PBC, and behavioral intention than the poor group. The concentration curve was significant for SN, PBC, and intention. In addition, the role of cognitive determinants in predicting cancer screening uptake has been confirmed in several studies [22–27]. Thus, it is expected that the better condition of cognitive determinants in the rich group of society will encourage more cancer screening uptake among them. Furthermore, our study found that people who had received at least one cancer screening test had higher average scores in SN, PBC, and intention compared to those who had not been screened. Intention was identified as the most important factor in predicting cancer screening behaviors. However, there was no significant difference in average attitude scores between individuals with a history of screening and those without.

The SN, as explained in the theoretical framework, represents a person’s belief about whether important individuals approve of a specific behavior [17]. This means that individuals who receive approval or encouragement from significant others to undergo cancer screening are more likely to actually do so. A study by Jalilian and Emdadi in Hamadan, Iran, found that subjective norms strongly influenced women’s decision to undergo cervical cancer screening [28]. This suggests that Iranians are influenced by subjective norms from people like their spouses, family, friends, and healthcare providers when it comes to cancer screening. However, the study also revealed that there is disparity in subjective norms, with wealthier individuals scoring higher in this area. This highlights the need to focus on promoting subjective norms, especially among vulnerable groups in society. Educational campaigns targeted at these groups could help reduce this disparity.

An important finding in the present study was the apparent disparity in PBC. In addition, our findings indicate that individuals with a history of cancer screening tend to have a higher PBC score. PBC refers to the degree to which a person feels how much control he or she has over whether or not to perform a behavior [17]. Lawal et al. reported a significant relationship between PBC and cancer screening uptake [29]. In line with our findings, Abamecha et al. in their study on 30–49 years old in Ethiopia reported PBC was intended to uptake cervical cancer screening [22]. Moreover, Roncancio et al. in their study indicated PBC was positively associated with the intention to be screened for cervical cancer [30]. Based on our results the intervention strategies in order to increase a person’s sense of control over cancer screening conditions may be usefulness of the results in order to cancer screening uptake. The development and implementation of PBC promotion campaigns focusing on disadvantaged people can lead to significant findings in reducing cancer screening disparity. Some effective methods for behavior change in this situation include self-monitoring (keeping track of behaviors), planning coping responses (identifying obstacles and ways to overcome them), and goal setting (planning what to do) [31]. These methods can be utilized in creating interventions.

In our study, we also looked into how socio-demographic factors are connected to disparities in cancer screening rates (Table 2). Our findings indicated the SES with 74.64% had the largest contribution among all determinants in increasing disparity in cancer screening uptake. In line with the findings of the present study, several studies have shown that SES predicts cancer screening uptake. Consistent with our findings, Calo et al., in their study in Houston, Texas, showed that people living in more socioeconomically deprived areas were less likely to use cancer screening services [32]. It should be noted that low SES has a direct negative effect on cancer prognosis [33]. Also, SES-related disparity can have an effect on cancer survival due to the effect on follow-up and access to treatment [34]. These points can be a warning to health policymakers and it highlights the need for more attention in disadvantaged groups. In Iran, free screenings for cervical, breast, and colon cancer are available [8]. Our findings indicated disparities in socioeconomic status when it comes to screening. One way to address this disparity is by offering free cancer screenings for high-risk and economically disadvantaged populations in Iran. This is a solution that health policy-makers could consider. Furthermore, Health insurance can cover the cost of cancer screening services, making it more accessible to a wider range of people without financial concerns.

Gender was the second important factor in increasing disparity with 22.25%. Women were 8.63 (6.87, 10.84) times more likely to be screened than men. In line with our findings, several studies also showed that cancer screening uptake was more common among women than men [13, 35, and 36]. The incidence and mortality rate of cancer is higher in men than in women; although this disparity is mainly due to their poor use of primary prevention strategies such as cancer screening uptake [37]. These findings especially emphasize the need for health promotion interventions among men. Thus, it seems that the development and implementation of campaigns to promote cancer screening uptake, especially among men, should be placed as a priority in health programs.

The odds ratio of screening for single people was 0.72 (CI OR: 0.53, 0.97), which shows that single people did screening significantly less than married people. On the other hand, widowed people had 1.71 (CI OR: 1.16, 2.53) times more chance of screening than married people. More cancer screening uptake among married people compared to single people has been shown in several studies [38, 39]. These findings highlight important points regarding the provision of screening programs and show the necessity of developing interventions for single people.

Our findings also showed that the proportion of performing cancer screening behaviors is higher among people with a positive family history of cancer. Several studies reported a higher chance of developing cancer among people with a family history [40, 41]. Furthermore, Ramsey et al. state that a significant proportion of people in the United States who have an immediate family member with cancer may be eligible for early or more aggressive cancer screening services [42]. The research conducted in the west of Iran indicates that the positive history of cancer in the family is related to the increase in the chance of performing cancer screening uptake [13, 27]. Family history assessment becomes important as a potential public health tool to help determine susceptibility to common cancers and can be placed on the agenda, especially for vulnerable groups.

### Study strengths and limitations

The present study has limitations that can be mentioned as follows. First, this study was conducted only among the urban population of Kermanshah, in the west of Iran, so it may not be generalizable to other people in Iran. Second, some important variables such as the frequency of cancer screenings test uptake were not examined and only the history of uptake was measured as yes or no. Third, the data collection was by self-declaration, which may not be accurately reported due to social desirability bias or recall biases and may be associated with a percentage of error [43]. Fourth, since this study was cross-sectional, care must be taken when interpreting the results because it does not examine causality. Fifth, the data collection for this study focused on individuals aged 30 and older, which aligns with the age range for many screenings. However, this limitation suggests the need for future research to gather more specific data on the recommended age group for screening for each type of cancer. Moreover, it is important to mention that this data was collected before the COVID-19 pandemic. There may be changes in the health beliefs of the community or the rates of cancer screenings now. As previously stated, this study was cross-sectional. There is a requirement for more thorough and current research. Finally, when it comes to free population-based screening for breast and cervical cancer, it is reasonable to expect that more women will undergo screenings. Therefore, the types of cancers being studied are not appropriate for comparison between men and women. This limitation should be acknowledged in the current study. Nevertheless, the present study has significant findings regarding cancer screening behaviors and disparity in western Iran and provides the basis for planning before the development of health promotion programs.

## Conclusion

To our knowledge, this is the first study in Iran that measures the disparity in cognitive determinants for cancer screening uptake. The findings can be very useful for health policymakers in Iran and highlight the importance of developing, implementing, and evaluating campaigns to promote PBC and SN, especially in disadvantaged people, which can help in reducing the disparity of cancer screening. Moreover, according to the results, in order to reduce cancer screening disparity, men, single people, and economically disadvantaged groups of Iranian society should be prioritized in health promotion programs. This knowledge can be used to develop a theory-driven and evidence-based intervention to increase cancer screening uptake and reduce disparities.

## Data Availability

The datasets used and/or analysed during the current study available from the corresponding author on reasonable request.
